# Targeting the lung endothelial niche to promote angiogenesis and regeneration: A review of applications

**DOI:** 10.3389/fmolb.2022.1093369

**Published:** 2022-12-19

**Authors:** Savas T. Tsikis, Thomas I. Hirsch, Scott C. Fligor, Mikayla Quigley, Mark Puder

**Affiliations:** Vascular Biology Program, Department of Surgery, Boston Children’s Hospital, Harvard Medical School, Boston, MA, United States

**Keywords:** endothelial niche, pulmonary hypoplasia, bronchopulmonar dysplasia, lung injury, translational outcomes

## Abstract

Lung endothelial cells comprise the pulmonary vascular bed and account for the majority of cells in the lungs. Beyond their role in gas exchange, lung ECs form a specialized microenvironment, or niche, with important roles in health and disease. In early development, progenitor ECs direct alveolar development through angiogenesis. Following birth, lung ECs are thought to maintain their regenerative capacity despite the aging process. As such, harnessing the power of the EC niche, specifically to promote angiogenesis and alveolar regeneration has potential clinical applications. Here, we focus on translational research with applications related to developmental lung diseases including pulmonary hypoplasia and bronchopulmonary dysplasia. An overview of studies examining the role of ECs in lung regeneration following acute lung injury is also provided. These diseases are all characterized by significant morbidity and mortality with limited existing therapeutics, affecting both young children and adults.

## 1 Introduction

The lung is the most vascularized organ in the human body. Pulmonary endothelial cells (ECs) are the primary components of the vascular bed and are the interface between the alveoli and the pulmonary circulation (Huertas et al., 2018). Lung ECs are involved in essential functions including the delivery of oxygen, and removal of carbon dioxide and waste. Besides these fundamental functions, pulmonary ECs form a specialized microenvironment, referred to as the vascular niche (Mammoto and Mammoto, 2019). The role of the vascular niche in the dysregulated response to disease is increasingly being recognized as important for therapeutics in several respiratory diseases such as acute lung injury (ALI), acute respiratory distress syndrome (ARDS), pulmonary embolism, and pulmonary hypertension (Li et al., 2022).

Within the vascular niche, vascular progenitor ECs interact reciprocally with the alveolar epithelium through paracrine signaling to regulate lung development and regeneration (Hogan et al., 2014). At the embryonic stage of lung development, mesenchymal cells express high amounts of vascular endothelial growth factor (VEGF) (White et al., 2007), which activates VEGF receptor 2 (VEGFR2). Downstream signaling results in the formation of capillary networks in conjunction with alveolar budding (Karaman et al., 2018; Apte et al., 2019). Defective lung vascular development contributes to diseases such as pulmonary hypoplasia (PH), and bronchopulmonary dysplasia (BPD), the most common chronic lung disease in infancy (Stoll et al., 2015). After birth, alveolar capillary ECs continue to play a key role in regeneration as demonstrated in animal models of pneumonectomy-induced compensatory lung growth (CLG) (Voswinckel et al., 2004; Sakurai et al., 2005). Angiogenesis driven by the elaborate signaling within the niche remains the key mechanism in this post-developmental lung regeneration (Ackermann et al., 2014). Therefore, in addition to infant diseases, targeting the vascular EC niche to promote angiogenesis, may have potential clinical applications in the recovery from ALI and COVID-19 ARDS which depend, in part, on alveolar regeneration (Medford and Millar, 2006).

**FIGURE 1:**
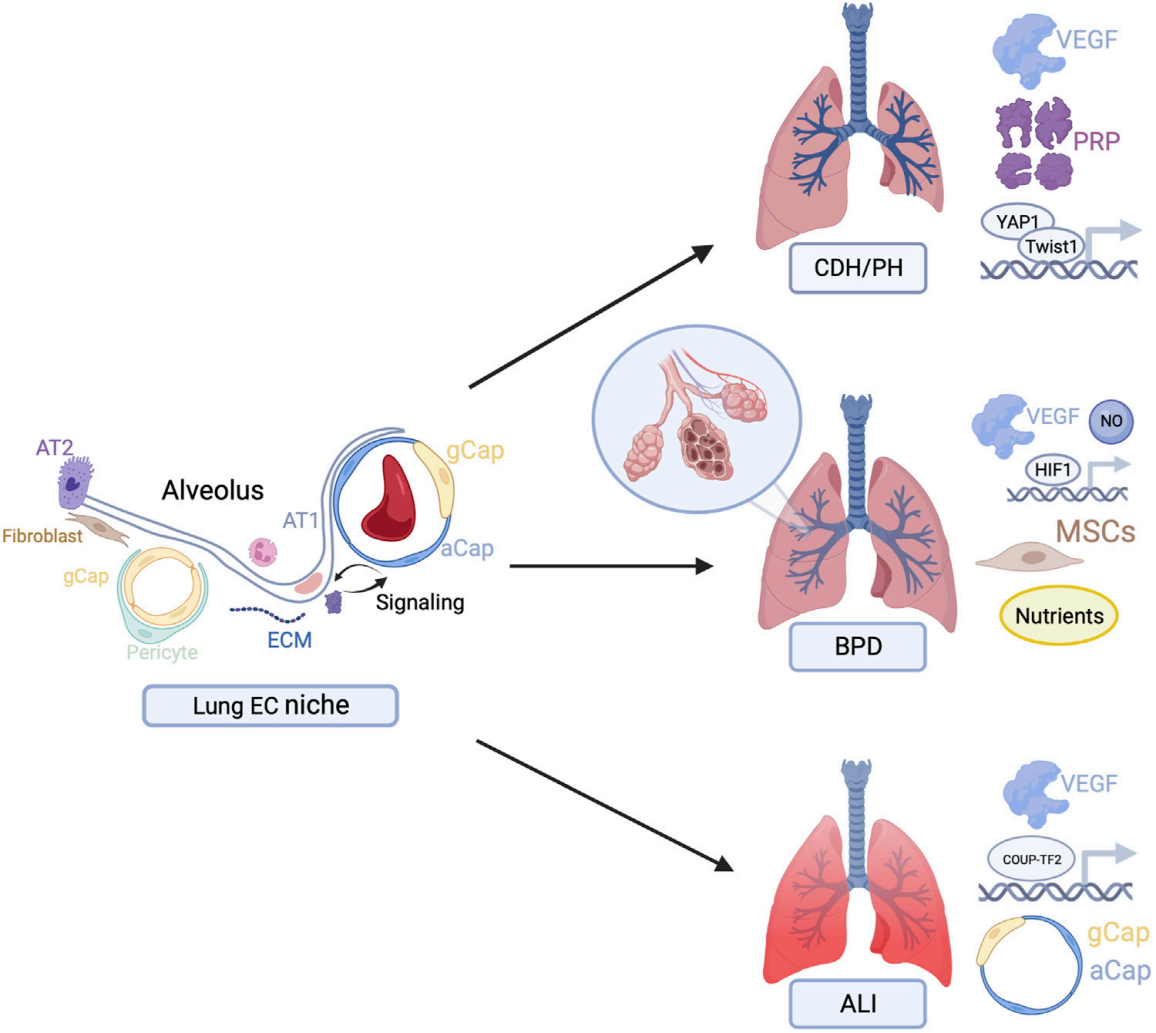
Overview of potential therapeutic targets to stimulate endothelial cell-driven angiogenesis in congenital diaphragmatic hernia (CDH), bronchopulmonary dysplasia (BPD), and acute lung injury (ALI). Figures created with BioRender.com.

Harnessing the power of the vascular EC niche specifically to promote angiogenesis and, by extension, alveolar growth and regeneration, may have important clinical implications. In this review, we focus on clinical applications for PH, BPD, and ALI (Figure 1). Given limited existing therapeutics for these conditions and their significant associated morbidity and mortality, there is an important need for targeted therapeutics.

## 2 Translational applications

### 2.1 Pulmonary hypoplasia

PH affects neonates, particularly those born with congenital diaphragmatic hernia (CDH). CDH occurs due to a developmental defect in the diaphragm resulting in the herniation of abdominal organs to the thoracic cavity *in utero*. In CDH, hypoplasia is frequently bilateral and is characterized by decreased branching of the bronchioles and acinar hypoplasia resulting in significant lung immaturity (Chandrasekharan et al., 2017). As a result, even after surgical repair, patients frequently require extensive periods of invasive cardiopulmonary support contributing to substantial mortality (up to 50%) (Seetharamaiah et al., 2009). Animal models of CLG following unilateral pneumonectomy have been used to study pulmonary hypoplastic diseases, including CDH given similarities in molecular patterning (Hsia, 2004). Therapeutics that accelerate lung growth by targeting the EC niche may prove promising in improving outcomes for infants with PH.

#### 2.1.1 Growth factors

CDH-associated PH is characterized by deficiency of VEGF within the vascular EC niche in both humans and animal models, particularly during alveologenesis (Chang et al., 2004; van der Horst et al., 2011). Exogenous VEGF administration accelerates lung growth in murine and porcine CLG models through VEGFR2-dependent signaling in ECs and increased alveolar units (Dao et al., 2018a; Dao et al., 2018b). In human clinical trials, VEGF has been evaluated as a therapeutic for cardiac ischemia, but side effects, particularly hypotension, have limited its clinical applicability (Crafts et al., 2015). Critically, as patients with severe CDH are often managed with cardiopulmonary support, hypotension could be mitigated with the support of the circuit. Clinical trials examining the role of VEGF as a therapeutic to accelerate lung growth in PH are warranted.

Pigment epithelium-derived factor (PEDF) has anti-angiogenic properties. Within the lung, VEGF/PEDF ratios correlate with tissue neovascularization and angiogenesis (Chetty et al., 2015). Roxadustat is a prolyl hydroxylase inhibitor that stabilizes hypoxia inducible factor alpha (HIF-α). In the murine CLG model, administration of roxadustat accelerated lung growth, improved lung function, and increased alveolarization through PEDF downregulation (Ko et al., 2020). Highlighting the impact of roxadustat throughout the niche, both ECs and alveolar epithelial cells demonstrated decreased PEDF expression *in vitro* (Ko et al., 2021)*.* Roxadustat has already been approved by the European Commission for the treatment of chronic kidney disease-associated anemia (Chen et al., 2019). Future work should focus on large animal models and other therapeutics with anti-PEDF effects.

#### 2.1.2 Platelet-rich plasma

Platelet-rich plasma (PRP) extract contains abundant angiopoietin-1 and multiple other angiogenic factors. Given that *in vivo* angiogenesis is promoted through multiple signaling factors, rather than a single growth factor, PRP is hypothesized to have improved effects on organ regeneration (Benest et al., 2006). PRP increased phosphorylation of the angiogenic factor receptor Tie2, accelerated CLG following murine left pneumonectomy, and increased epithelial cell budding *in vitro* (Mammoto et al., 2016). Since PRP can be generated through simple methods and preserved for extended periods, these results suggest a potential role in improving outcomes for neonates with PH and CDH, although more investigation is needed.

#### 2.1.3 Transcription factors

The Hippo signaling transducer, Yes-associated protein (YAP) 1, binds to the TEA domain transcription factor and plays a role in organ growth and regeneration (Ota and Sasaki, 2008). Mammoto et al. demonstrated that YAP1 stimulates endothelial cell sprouting and alveolar epithelial morphogenesis *in vitro* and promotes CLG following unilateral pneumonectomy in mice through the angiopoietin-Tie2 pathway *in vivo* (Mammoto et al., 2019a). Twist1 is a transcription factor, that contributes to age-mediated declines in angiogenesis (Li et al., 2014). While Twist1 decreases lung growth in aged mice following pneumonectomy, in young ECs, Twist1 overexpression is associated with increased EC proliferation and migration, along with concomitant increases in VEGFR2 expression (Hendee et al., 2021). Both YAP1 and Twist1 also sense mechanical forces, including shear stress and extracellular matrix (ECM) stiffness which control vascular formation and function (Mammoto et al., 2009; Wei et al., 2015; Mammoto et al., 2019b). YAP1 and Twist1 are thus potential therapeutic targets for CDH-associated PH in young patients dependent on lung regeneration, although more research is needed.

### 2.2 Bronchopulmonary dysplasia

BPD is a chronic lung disease of premature newborns that is characterized by impaired development of lung parenchyma and respiratory microvasculature (Jobe, 1999; Coalson, 2003). Improving management of preterm neonates has resulted in an altered disease phenotype. The impaired lung development of ‘new’ BPD is due to multiple perinatal factors, including infection, inflammation and disorganized repair processes resulting in a paucity of alveoli and a dysmorphic capillary network (Husain et al., 1998; Thibeault et al., 2000; Coalson, 2003; Appuhn et al., 2021).

The conserved pattern of histological and vascular signaling abnormalities in new BPD led to the hypothesis that ECs and aberrant signaling drive disease (Abman, 2001). A common preclinical model for investigating BPD is the rodent hyperoxia-induced lung injury model, where newborn animals are exposed to hyperoxia to impair lung development. The lung endothelial niche provides an upstream target for reversing the impaired alveologenesis in BPD. Preserved angiogenesis facilitates alveolarization and lung growth, translating to functional improvement (Appuhn et al., 2021; Vohwinkel and Tuder, 2021). Preclinical investigations have evaluated several EC niche mediators, stem cells, and nutrients.

#### 2.2.1 Growth factors

VEGF is central to the vascular hypothesis of BPD and may have a potential role in treatment (Abman, 2001). In hyperoxia-challenged mice, administration of anti-angiogenic agents impaired angiogenesis and alveolarization (Tang et al., 2012; Wallace et al., 2018), while over-expression of VEGF attenuated the adverse effects of hyperoxia on alveolarization (Alvira, 2016). In another study in hyperoxia-exposed Sprague-Dawley rats, recombinant human VEGF treatment reversed the BPD phenotype and accelerated EC-mediated vessel growth and alveolarization (Kunig et al., 2005). In lung development, growth factors released from the niche coordinate epithelial cell growth and are critical for mediating the development of distal airspace structures (Abman, 2001). Furthermore VEGFR2 signaling promotes differentiation of alveolar type II epithelial cells (AT2) and stimulates secretion of surfactant, which are critical in BPD (Cross et al., 2003). There has also been success targeting antagonists of VEGF. In a recent study, rats administered anti-soluble fms-like tyrosine kinase-1 monoclonal antibody, which inhibits an endogenous antagonist to VEGF, improved radial alveolar count, vessel density, and lung function (Wallace et al., 2018).

Hepatocyte growth factor (HGF) is a downstream mediator of VEGF with evidence of stimulating angiogenesis and alveolar epithelial cell proliferation in murine hyperoxia-induced BPD. HGF specifically stimulates epithelial cell migration and stimulates fibroblasts to accelerate healing of alveolar epithelial cells after lung injury (Ito et al., 2014). Insulin growth factor-1 (IGF-1) is another downstream mediator of VEGF. Clinically, decreased postnatal IGF-1 concentrations were associated with an increased risk of BPD (Lofqvist et al., 2012). Preclinical interventional studies in rats provided evidence that restoring IGF-1 concentration could prevent or treat BPD (Jin et al., 2007). This led to a phase II randomized controlled trial that found IGF-1 infusions after preterm birth decreased severe BPD by 53% (Ley et al., 2019).

#### 2.2.2 Nitric oxide (NO)

NO is gaseous signaling molecule that is a direct downstream mediator of VEGF (Ziche et al., 1997). VEGF stimulates NO production *via* expression of endothelial nitric oxide synthase (eNOS) (Seedorf et al., 2016). In both *ex vivo* (Muehlethaler et al., 2008) and *in vivo* (Tang et al., 2004) studies, administration of NO improved lung growth after VEGF inhibition. NO also improved lung growth in a postnatal hyperoxia model (Lin et al., 2005). Mechanistically, there is evidence that shear stress increases eNOS activity and the production of NO through PI3 kinase/Akt signaling in the niche (Kumar et al., 2010). Sheer stress may occur in BPD secondary to alveolar collapse at low lung ventilation volumes (Kalikkot Thekkeveedu et al., 2017).

#### 2.2.3 HIF

HIFs are potent upstream regulators of VEGF and angiogenesis (Kunig et al., 2005). In an oxygen poor environment, HIF degradation is inhibited, increasing HIF levels increase transcription of genes involved in angiogenesis. Prolyl hydroxylases target HIF for degradation in oxygen rich environments, negatively regulating expression of angiogenesis-related genes (Semenza, 2014). In a study on endotoxin-treated premature rats, administration of prolyl hydroxylase inhibitor, dimethyloxalylglycine or GSK360A, *via* the intraperitoneal route for 2 weeks preserved lung alveolar and vascular growth and lung function with potential applications for BPD (Hirsch et al., 2020).

#### 2.2.4 Mesenchymal stem cells (MSCs)

MSCs are fibroblast-like shaped cells residing in mesodermal tissues that have pro-angiogenic, anti-inflammatory and tissue-regenerative potential (Mobius and Rudiger, 2016). MSCs are predominantly involved in BPD *via* paracrine effects, secreting VEGF to stimulate angiogenesis within the EC niche (Fung and Thebaud, 2014). Rodent hyperoxia-induced lung injury models have demonstrated the preventative effect of both intratracheal and intravenous administration of Bone Marrow MSCs (BM-MSCs) (Aslam et al., 2009; van Haaften et al., 2009). While most of the work has been done in rodent models, results from a phase I clinical trial using MSCs from umbilical cord blood in infants at risk for BPD have been promising (Chang et al., 2014).

#### 2.2.5 Nutrients

L-citrulline, vitamin D, and vitamin A are nutrients that act on ECs to improve BPD in preclinical models (Ma et al., 2017). Multiple rodent models of hyperoxia-induced BPD have demonstrated attenuation of pulmonary injury after administration of L-citrulline (Grisafi et al., 2012; Sopi et al., 2012). L-citrulline acts *via* enhancement of VEGF and eNOS expression and reversal of airway relaxation associated with hyperoxia (Sopi et al., 2012). Endotoxin-exposed rats were used to study antenatal administration of vitamin D. After 14 days, lung harvest and analysis provided evidence for vitamin D as a pro-angiogenic mediator of embryonic ECs, leading to growth and tube formation (Mandell et al., 2014). Vitamin A is the only nutrient currently recommended in BPD, after preclinical evidence in preterm lambs demonstrated its ability to increase alveolar capillary growth through increased VEGF expression and induction of alveolar septation (Albertine et al., 2010).

### 2.3 Lung injury

ALI and ARDS are characterized by the onset of bilateral lung infiltrates with alveolar injury, damage to the alveolar-capillary membrane, and leakage of protein-rich exudate resulting in severe hypoxemia and systemic dysfunction (Fan et al., 2018). Persistent lung inflammation and remodeling following the initial insult are also linked to diminished pulmonary functional outcome in long-term studies of animals and humans (de Souza Xavier Costa et al., 2017; Carfi et al., 2020). There are currently no effective medications targeting the underlying pathophysiology of ALI and the long-term response to injury.

Targeting the lung EC niche may have therapeutic applications in ALI. Lung ECs have the potential to accelerate repair of damaged alveoli through autocrine and paracrine signaling to the surrounding alveolar epithelium and mesenchyme (Li et al., 2022). Recent evidence further suggests that the native lung endothelium retains substantial regenerative capacity and is the source of neovascularization after influenza injury (Zhao et al., 2020). Therefore there are several important targets for potential therapeutics.

#### 2.3.1 Angiogenic growth factors

VEGF has been evaluated in animal ALI models but the data regarding its role as a therapeutic agent have been conflicting (Medford and Millar, 2006). In the short-term, VEGF may have pathologic effects by increasing alveolar-capillary membrane permeability within the EC niche and contributing to exudate formation (Tomita et al., 2020). For example, SU5416, a potent and selective VEGFR2 inhibitor, ameliorated epithelial cell injury and histopathological changes in a murine ALI model following lipopolysaccharide (LPS) administration (Huang et al., 2019). In this study, SU5416 further suppressed the immune response within the niche by decreasing neutrophil cell population and proinflammatory cytokines. However, VEGF may also act as a pneumotrophic factor on alveolar epithelial cells and stimulate endothelial-mediated angiogenesis, facilitating lung recovery and regeneration in the long-term (Kasahara et al., 2000; Ohwada et al., 2003). More recent studies have demonstrated a correlation between changes in lung function with decreased vascularization, VEGF expression, and VEGFR2 activation in the long-term following injury (Tsikis et al., 2022). Furthermore, in human ARDS lung samples, VEGF expression was negatively correlated with EC apoptosis and was not dependent on changes in the population of AT2 (Abadie et al., 2005). Overall, these data highlight the potential role for VEGF administration in the regenerative and recovery phases of lung injury. Agents such as roxadustat, that can upregulate endogenous VEGF expression, or the provision of multiple pro-angiogenic growth factors (e.g., PRP) have potential clinical applicability. More exploratory studies are needed at the preclinical level to further elucidate the role of angiogenic growth factors in the recovery from ALI.

#### 2.3.2 Transcription factors

Transcription factors implicated in developmental angiogenesis may be involved in reversing cellular senescence and promoting lung regeneration after severe lung injury. In one study, Zhao et al. demonstrated that chicken ovalbumin upstream promoter-transcription factor 2 (COUP-TF2) drives EC proliferation and migration in part by enhancing the VEGFA/VEGFR2 pathway (Zhao et al., 2020). Proinflammatory cytokines interleukin 1β (IL-1β) and tumor necrosis factor-α (TNF-α) that are strongly expressed in the vascular niche in response to injury suppressed COUP-TF2 expression, while COUP-TF2 ablation exacerbated influenza lung injury (Zhao et al., 2020). These results suggest that stabilization of COUP-TF2 may represent a therapeutic strategy to enhance recovery from ALI, however more work is needed to further elucidate this role.

#### 2.3.3 Specialized cells

Recent single-cell studies have revealed two distinct populations of capillary ECs; aerocyte cells (aCap), marked by the expression of carbonic anhydrase 4 (Car4), and general cells (gCap) (Gillich et al., 2020; Vila Ellis et al., 2020). Aerocytes are large cells, similar to alveolar type I epithelial cells (AT1), specialized for gas exchange and leukocyte trafficking. AT1-derived *Vegfa* expression of VEGF 188 drives the specification of aCap ECs during alveolar development (Fidalgo et al., 2022). The interface created between aCap and AT1 cells within the niche may be important in ALI (Gillich et al., 2020). In one study, Car4^+^ ECs were highly concentrated in the most damaged alveoli following influenza-induced ALI and pseudo-time analysis of single EC transcriptomes suggested that these cells are highly proliferative following injury (Niethamer et al., 2020). Ligand-receptor analysis confirmed crosstalk between Car4+ ECs and other components of the vascular niche, such as AT1 cells and with collagens, integrins, and metalloproteases in the ECM (Niethamer et al., 2020). In adulthood, gCap cells maintain the alveolar endothelium and may function as progenitor cells as during lung regeneration (Gillich et al., 2020).

Harvesting these specialized ECs and further characterizing their interactions within the niche can have therapeutic applications. Future investigations should focus on translating these findings to a human therapeutic. Limitations such as unintended effects from vascular EC proliferation should also be explored.

## 3 Discussion

Throughout lung development, angiogenesis occurs in parallel with alveolarization, and ECs play an important role in orchestrating growth by signaling to the surrounding non-vascular parenchyma. Recent work further suggests that the EC niche contributes to lung regeneration in adulthood after injury. Despite this growing body of evidence, the extent of lung EC heterogeneity and their function in regeneration remains incompletely understood.

Harvesting the power of the EC niche to stimulate angiogenesis may have important clinical applications (Figure 1). Recent studies suggest that growth factors, including VEGF and related signaling mediators, may have important therapeutic applications in CDH, BPD, and ALI. At the gene level, transcription factors have been implicated in reversing EC senescence. Finally, specialized cells such as MSCs, and aCap/gCap cells have been discovered as pro-angiogenic mediators in BPD and ALI animal models, respectively.

In this review, we elected to limit detailed description of the underlying molecular mechanisms and have instead focused on the translational applicability for the various therapeutic targets. Future efforts should be directed on bridging these findings to novel therapeutics that promote angiogenesis and lung regeneration. For example, mRNA technology can be utilized for targeted gene expression and has important potential in the lung where directed delivery through inhalation or nebulization can be achieved (Bhat et al., 2021). Such research would have clinical applications for lung diseases described in this review, which are characterized by significant mortality and limited therapeutics.

## References

[B1] Abadie Y. Bregeon F. Papazian L. Lange F. Chailley-Heu B. Thomas P. (2005). Decreased VEGF concentration in lung tissue and vascular injury during ARDS. Eur. Respir. J. 25 (1), 139–146. 10.1183/09031936.04.00065504 15640335

[B2] Abman S. H. (2001). Bronchopulmonary dysplasia: "a vascular hypothesis. Am. J. Respir. Crit. Care Med. 164 (10), 1755–1756. 10.1164/ajrccm.164.10.2109111c 11734417

[B3] Ackermann M. Houdek J. P. Gibney B. C. Ysasi A. Wagner W. Belle J. (2014). Sprouting and intussusceptive angiogenesis in postpneumonectomy lung growth: Mechanisms of alveolar neovascularization. Angiogenesis 17 (3), 541–551. 10.1007/s10456-013-9399-9 24150281 PMC4061467

[B4] Albertine K. H. Dahl M. J. Gonzales L. W. Wang Z. M. Metcalfe D. Hyde D. M. (2010). Chronic lung disease in preterm lambs: Effect of daily vitamin A treatment on alveolarization. Am. J. Physiol. Lung Cell Mol. Physiol. 299 (1), L59–L72. 10.1152/ajplung.00380.2009 20382748 PMC2904099

[B5] Alvira C. M. (2016). Aberrant pulmonary vascular growth and remodeling in bronchopulmonary dysplasia. Front. Med. (Lausanne) 3, 21. 10.3389/fmed.2016.00021 27243014 PMC4873491

[B6] Appuhn S. V. Siebert S. Myti D. Wrede C. Surate Solaligue D. E. Perez-Bravo D. (2021). Capillary changes precede disordered alveolarization in a mouse model of bronchopulmonary dysplasia. Am. J. Respir. Cell Mol. Biol. 65 (1), 81–91. 10.1165/rcmb.2021-0004OC 33784484

[B7] Apte R. S. Chen D. S. Ferrara N. (2019). VEGF in signaling and disease: Beyond discovery and development. Cell 176 (6), 1248–1264. 10.1016/j.cell.2019.01.021 30849371 PMC6410740

[B8] Aslam M. Baveja R. Liang O. D. Fernandez-Gonzalez A. Lee C. Mitsialis S. A. (2009). Bone marrow stromal cells attenuate lung injury in a murine model of neonatal chronic lung disease. Am. J. Respir. Crit. Care Med. 180 (11), 1122–1130. 10.1164/rccm.200902-0242OC 19713447 PMC2784417

[B9] Benest A. V. Salmon A. H. Wang W. Glover C. P. Uney J. Harper S. J. (2006). VEGF and angiopoietin-1 stimulate different angiogenic phenotypes that combine to enhance functional neovascularization in adult tissue. Microcirculation 13 (6), 423–437. 10.1080/10739680600775940 16864410

[B10] Bhat B. Karve S. Anderson D. G. (2021). mRNA therapeutics: Beyond vaccine applications. Trends Mol. Med. 27 (9), 923–924. 10.1016/j.molmed.2021.05.004 34172390

[B11] Carfi A. Bernabei R. Landi F. Gemelli Against C.-P.-A. C. S. G. (2020). Persistent symptoms in patients after acute COVID-19. JAMA 324 (6), 603–605. 10.1001/jama.2020.12603 32644129 PMC7349096

[B12] Chandrasekharan P. K. Rawat M. Madappa R. Rothstein D. H. Lakshminrusimha S. (2017). Congenital Diaphragmatic hernia - a review. Matern. Health Neonatol. Perinatol. 3, 6. 10.1186/s40748-017-0045-1 28331629 PMC5356475

[B13] Chang R. Andreoli S. Ng Y. S. Truong T. Smith S. R. Wilson J. (2004). VEGF expression is downregulated in nitrofen-induced congenital diaphragmatic hernia. J. Pediatr. Surg. 39 (6), 825–828. 10.1016/j.jpedsurg.2004.02.015 15185205

[B14] Chang Y. S. Ahn S. Y. Yoo H. S. Sung S. I. Choi S. J. Oh W. I. (2014). Mesenchymal stem cells for bronchopulmonary dysplasia: Phase 1 dose-escalation clinical trial. J. Pediatr. 164 (5), 966–972. 10.1016/j.jpeds.2013.12.011 24508444

[B15] Chen N. Hao C. Liu B. C. Lin H. Wang C. Xing C. (2019). Roxadustat treatment for anemia in patients undergoing long-term dialysis. N. Engl. J. Med. 381 (11), 1011–1022. 10.1056/NEJMoa1901713 31340116

[B16] Chetty A. Bennett M. Dang L. Nakamura D. Cao G. J. Mujahid S. (2015). Pigment epithelium-derived factor mediates impaired lung vascular development in neonatal hyperoxia. Am. J. Respir. Cell Mol. Biol. 52 (3), 295–303. 10.1165/rcmb.2013-0229OC 25054647 PMC4370256

[B17] Coalson J. J. (2003). Pathology of new bronchopulmonary dysplasia. Semin. Neonatol. 8 (1), 73–81. 10.1016/s1084-2756(02)00193-8 12667832

[B18] Crafts T. D. Jensen A. R. Blocher-Smith E. C. Markel T. A. (2015). Vascular endothelial growth factor: Therapeutic possibilities and challenges for the treatment of ischemia. Cytokine 71 (2), 385–393. 10.1016/j.cyto.2014.08.005 25240960

[B19] Cross M. J. Dixelius J. Matsumoto T. Claesson-Welsh L. (2003). VEGF-receptor signal transduction. Trends Biochem. Sci. 28 (9), 488–494. 10.1016/S0968-0004(03)00193-2 13678960

[B20] Dao D. T. Anez-Bustillos L. Pan A. O'Loughlin A. A. Mitchell P. D. Fell G. L. (2018a). Vascular endothelial growth factor enhances compensatory lung growth in piglets. Surgery 164 (6), 1279–1286. 10.1016/j.surg.2018.07.003 30193736 PMC6446901

[B21] Dao D. T. Nandivada P. Vuong J. T. Anez-Bustillos L. Pan A. Kishikawa H. (2018b). Vascular endothelial growth factor accelerates compensatory lung growth by increasing the alveolar units. Pediatr. Res. 83 (6), 1182–1189. 10.1038/pr.2018.41 29638228 PMC6019135

[B22] de Souza Xavier Costa N. Ribeiro Junior G. Dos Santos Alemany A. A. Belotti L. Zati D. H. Frota Cavalcante M. (2017). Early and late pulmonary effects of nebulized LPS in mice: An acute lung injury model. PLoS One 12 (9), e0185474. 10.1371/journal.pone.0185474 28953963 PMC5617199

[B23] Fan E. Brodie D. Slutsky A. S. (2018). Acute respiratory distress syndrome: Advances in diagnosis and treatment. JAMA 319 (7), 698–710. 10.1001/jama.2017.21907 29466596

[B24] Fidalgo M. F. Fonseca C. G. Caldas P. Raposo A. A. Balboni T. Henao-Misikova L. (2022). Aerocyte specification and lung adaptation to breathing is dependent on alternative splicing changes. Life Sci. Alliance 5 (12), e202201554. 10.26508/lsa.202201554 36220570 PMC9554796

[B25] Fung M. E. Thebaud B. (2014). Stem cell-based therapy for neonatal lung disease: It is in the juice. Pediatr. Res. 75 (1-1), 2–7. 10.1038/pr.2013.176 24126817 PMC3940470

[B26] Gillich A. Zhang F. Farmer C. G. Travaglini K. J. Tan S. Y. Gu M. (2020). Capillary cell-type specialization in the alveolus. Nature 586 (7831), 785–789. 10.1038/s41586-020-2822-7 33057196 PMC7721049

[B27] Grisafi D. Tassone E. Dedja A. Oselladore B. Masola V. Guzzardo V. (2012). L-citrulline prevents alveolar and vascular derangement in a rat model of moderate hyperoxia-induced lung injury. Lung 190 (4), 419–430. 10.1007/s00408-012-9382-z 22430123

[B28] Hendee K. Hunyenyiwa T. Matus K. Toledo M. Mammoto A. Mammoto T. (2021). Twist1 signaling in age-dependent decline in angiogenesis and lung regeneration. Aging (Albany NY) 13 (6), 7781–7799. 10.18632/aging.202875 33764901 PMC8034921

[B29] Hirsch K. Taglauer E. Seedorf G. Callahan C. Mandell E. White C. W. (2020). Perinatal hypoxia-inducible factor stabilization preserves lung alveolar and vascular growth in experimental bronchopulmonary dysplasia. Am. J. Respir. Crit. Care Med. 202 (8), 1146–1158. 10.1164/rccm.202003-0601OC 32551816 PMC7560790

[B30] Hogan B. L. Barkauskas C. E. Chapman H. A. Epstein J. A. Jain R. Hsia C. C. (2014). Repair and regeneration of the respiratory system: Complexity, plasticity, and mechanisms of lung stem cell function. Cell Stem Cell 15 (2), 123–138. 10.1016/j.stem.2014.07.012 25105578 PMC4212493

[B31] Hsia C. C. (2004). Signals and mechanisms of compensatory lung growth. J. Appl. Physiol. 97 (5), 1992–1998. 10.1152/japplphysiol.00530.2004 15475557

[B32] Huang X. Zhu J. Jiang Y. Xu C. Lv Q. Yu D. (2019). SU5416 attenuated lipopolysaccharide-induced acute lung injury in mice by modulating properties of vascular endothelial cells. Drug Des. Devel Ther. 13, 1763–1772. 10.2147/DDDT.S188858 PMC6536715 31213766

[B33] Huertas A. Guignabert C. Barbera J. A. Bartsch P. Bhattacharya J. Bhattacharya S. (2018). Pulmonary vascular endothelium: The orchestra conductor in respiratory diseases: Highlights from basic research to therapy. Eur. Respir. J. 51 (4), 1700745. 10.1183/13993003.00745-2017 29545281

[B34] Husain A. N. Siddiqui N. H. Stocker J. T. (1998). Pathology of arrested acinar development in postsurfactant bronchopulmonary dysplasia. Hum. Pathol. 29 (7), 710–717. 10.1016/s0046-8177(98)90280-5 9670828

[B35] Ito Y. Correll K. Schiel J. A. Finigan J. H. Prekeris R. Mason R. J. (2014). Lung fibroblasts accelerate wound closure in human alveolar epithelial cells through hepatocyte growth factor/c-Met signaling. Am. J. Physiol. Lung Cell Mol. Physiol. 307 (1), L94–L105. 10.1152/ajplung.00233.2013 24748602 PMC4080284

[B36] Jin Z. A. Jin Z. Y. Chi Y. X. Lu J. R. (2007). Effects of recombinant human insulin-like growth factor-1 on the expression of Clara cell secretory protein in lung of hyperoxia-exposed newborn rats. Zhonghua Er Ke Za Zhi 45 (5), 369–373. 17697625

[B37] Jobe A. J. (1999). The new BPD: An arrest of lung development. Pediatr. Res. 46 (6), 641–643. 10.1203/00006450-199912000-00007 10590017

[B38] Kalikkot Thekkeveedu R. Guaman M. C. Shivanna B. (2017). Bronchopulmonary dysplasia: A review of pathogenesis and pathophysiology. Respir. Med. 132, 170–177. 10.1016/j.rmed.2017.10.014 29229093 PMC5729938

[B39] Karaman S. Leppanen V. M. Alitalo K. (2018). Vascular endothelial growth factor signaling in development and disease. Development 145 (14), dev151019. 10.1242/dev.151019 30030240

[B40] Kasahara Y. Tuder R. M. Taraseviciene-Stewart L. Le Cras T. D. Abman S. Hirth P. K. (2000). Inhibition of VEGF receptors causes lung cell apoptosis and emphysema. J. Clin. Invest. 106 (11), 1311–1319. 10.1172/JCI10259 11104784 PMC387249

[B41] Ko V. H. Yu L. J. Dao D. T. Li X. Secor J. D. Anez-Bustillos L. (2020). Roxadustat (FG-4592) accelerates pulmonary growth, development, and function in a compensatory lung growth model. Angiogenesis 23, 637–649. 10.1007/s10456-020-09735-9 32666268

[B42] Ko V. H. Yu L. J. Secor J. D. Pan A. Mitchell P. D. Kishikawa H. (2021). Deficiency in pigment epithelium-derived factor accelerates pulmonary growth and development in a compensatory lung growth model. FASEB J. 35 (10), e21850. 10.1096/fj.202002661RR 34569654

[B43] Kumar S. Sud N. Fonseca F. V. Hou Y. Black S. M. (2010). Shear stress stimulates nitric oxide signaling in pulmonary arterial endothelial cells via a reduction in catalase activity: Role of protein kinase C delta. Am. J. Physiol. Lung Cell Mol. Physiol. 298 (1), L105–L116. 10.1152/ajplung.00290.2009 19897742 PMC2806197

[B44] Kunig A. M. Balasubramaniam V. Markham N. E. Morgan D. Montgomery G. Grover T. R. (2005). Recombinant human VEGF treatment enhances alveolarization after hyperoxic lung injury in neonatal rats. Am. J. Physiol. Lung Cell Mol. Physiol. 289 (4), L529–L535. 10.1152/ajplung.00336.2004 15908474

[B45] Ley D. Hallberg B. Hansen-Pupp I. Dani C. Ramenghi L. A. Marlow N. (2019). rhIGF-1/rhIGFBP-3 in preterm infants: A phase 2 randomized controlled trial. J. Pediatr. 206, 56–65. 10.1016/j.jpeds.2018.10.033 30471715 PMC6389415

[B46] Li J. Liu C. H. Sun Y. Gong Y. Fu Z. Evans L. P. (2014). Endothelial TWIST1 promotes pathological ocular angiogenesis. Invest. Ophthalmol. Vis. Sci. 55 (12), 8267–8277. 10.1167/iovs.14-15623 25414194 PMC4541480

[B47] Li Y. X. Wang H. B. Li J. Jin J. B. Hu J. B. Yang C. L. (2022). Targeting pulmonary vascular endothelial cells for the treatment of respiratory diseases. Front. Pharmacol. 13, 983816. 10.3389/fphar.2022.983816 36110525 PMC9468609

[B48] Lin Y. J. Markham N. E. Balasubramaniam V. Tang J. R. Maxey A. Kinsella J. P. (2005). Inhaled nitric oxide enhances distal lung growth after exposure to hyperoxia in neonatal rats. Pediatr. Res. 58 (1), 22–29. 10.1203/01.PDR.0000163378.94837.3E 15879297

[B49] Lofqvist C. Hellgren G. Niklasson A. Engstrom E. Ley D. Hansen-Pupp I. (2012). Low postnatal serum IGF-I levels are associated with bronchopulmonary dysplasia (BPD). Acta Paediatr. 101 (12), 1211–1216. 10.1111/j.1651-2227.2012.02826.x 22924869 PMC3569611

[B50] Ma L. Zhou P. Neu J. Lin H. C. (2017). Potential nutrients for preventing or treating bronchopulmonary dysplasia. Paediatr. Respir. Rev. 22, 83–88. 10.1016/j.prrv.2016.08.013 27843119

[B51] Mammoto A. Connor K. M. Mammoto T. Yung C. W. Huh D. Aderman C. M. (2009). A mechanosensitive transcriptional mechanism that controls angiogenesis. Nature 457 (7233), 1103–1108. 10.1038/nature07765 19242469 PMC2708674

[B52] Mammoto A. Mammoto T. (2019). Vascular niche in lung alveolar development, homeostasis, and regeneration. Front. Bioeng. Biotechnol. 7, 318. 10.3389/fbioe.2019.00318 31781555 PMC6861452

[B53] Mammoto T. Chen Z. Jiang A. Jiang E. Ingber D. E. Mammoto A. (2016). Acceleration of lung regeneration by platelet-rich plasma extract through the low-density lipoprotein receptor-related protein 5-tie2 pathway. Am. J. Respir. Cell Mol. Biol. 54 (1), 103–113. 10.1165/rcmb.2015-0045OC 26091161 PMC5455682

[B54] Mammoto T. Muyleart M. Mammoto A. (2019a). Endothelial YAP1 in regenerative lung growth through the angiopoietin-tie2 pathway. Am. J. Respir. Cell Mol. Biol. 60 (1), 117–127. 10.1165/rcmb.2018-0105OC 30156429 PMC6348720

[B55] Mammoto T. Torisawa Y. S. Muyleart M. Hendee K. Anugwom C. Gutterman D. (2019b). Effects of age-dependent changes in cell size on endothelial cell proliferation and senescence through YAP1. Aging (Albany NY) 11 (17), 7051–7069. 10.18632/aging.102236 31487690 PMC6756888

[B56] Mandell E. Seedorf G. Gien J. Abman S. H. (2014). Vitamin D treatment improves survival and infant lung structure after intra-amniotic endotoxin exposure in rats: Potential role for the prevention of bronchopulmonary dysplasia. Am. J. Physiol. Lung Cell Mol. Physiol. 306 (5), L420–L428. 10.1152/ajplung.00344.2013 24414254 PMC3949057

[B57] Medford A. R. Millar A. B. (2006). Vascular endothelial growth factor (VEGF) in acute lung injury (ALI) and acute respiratory distress syndrome (ARDS): Paradox or paradigm? Thorax 61 (7), 621–626. 10.1136/thx.2005.040204 16807391 PMC1828639

[B58] Mobius M. A. Rudiger M. (2016). Mesenchymal stromal cells in the development and therapy of bronchopulmonary dysplasia. Mol. Cell Pediatr. 3 (1), 18. 10.1186/s40348-016-0046-6 27142639 PMC4854850

[B59] Muehlethaler V. Kunig A. M. Seedorf G. Balasubramaniam V. Abman S. H. (2008). Impaired VEGF and nitric oxide signaling after nitrofen exposure in rat fetal lung explants. Am. J. Physiol. Lung Cell Mol. Physiol. 294 (1), L110–L120. 10.1152/ajplung.00407.2007 17993583

[B60] Niethamer T. K. Stabler C. T. Leach J. P. Zepp J. A. Morley M. P. Babu A. (2020). Defining the role of pulmonary endothelial cell heterogeneity in the response to acute lung injury. Elife 9, e53072. 10.7554/eLife.53072 32091393 PMC7176435

[B61] Ohwada A. Yoshioka Y. Iwabuchi K. Nagaoka I. Dambara T. Fukuchi Y. (2003). VEGF regulates the proliferation of acid-exposed alveolar lining epithelial cells. Thorax 58 (4), 328–332. 10.1136/thorax.58.4.328 12668796 PMC1746622

[B62] Ota M. Sasaki H. (2008). Mammalian Tead proteins regulate cell proliferation and contact inhibition as transcriptional mediators of Hippo signaling. Development 135 (24), 4059–4069. 10.1242/dev.027151 19004856

[B63] Sakurai M. K. Greene A. K. Wilson J. Fauza D. Puder M. (2005). Pneumonectomy in the mouse: Technique and perioperative management. J. Invest. Surg. 18 (4), 201–205. 10.1080/08941930591004485 16126631

[B64] Seedorf G. Metoxen A. J. Rock R. Markham N. Ryan S. Vu T. (2016). Hepatocyte growth factor as a downstream mediator of vascular endothelial growth factor-dependent preservation of growth in the developing lung. Am. J. Physiol. Lung Cell Mol. Physiol. 310 (11), L1098–L1110. 10.1152/ajplung.00423.2015 27036872 PMC4935471

[B65] Seetharamaiah R. Younger J. G. Bartlett R. H. Hirschl R. B. Congenital Diaphragmatic Hernia Study G. (2009). Factors associated with survival in infants with congenital diaphragmatic hernia requiring extracorporeal membrane oxygenation: A report from the congenital diaphragmatic hernia study group. J. Pediatr. Surg. 44 (7), 1315–1321. 10.1016/j.jpedsurg.2008.12.021 19573654

[B66] Semenza G. L. (2014). Oxygen sensing, hypoxia-inducible factors, and disease pathophysiology. Annu. Rev. Pathol. 9, 47–71. 10.1146/annurev-pathol-012513-104720 23937437

[B67] Sopi R. B. Zaidi S. I. Mladenov M. Sahiti H. Istrefi Z. Gjorgoski I. (2012). L-citrulline supplementation reverses the impaired airway relaxation in neonatal rats exposed to hyperoxia. Respir. Res. 13, 68. 10.1186/1465-9921-13-68 22870905 PMC3487946

[B68] Stoll B. J. Hansen N. I. Bell E. F. Walsh M. C. Carlo W. A. Shankaran S. (2015). Trends in care practices, morbidity, and mortality of extremely preterm neonates. JAMA 314 (10), 1039–1051. 10.1001/jama.2015.10244 26348753 PMC4787615

[B69] Tang J. R. Karumanchi S. A. Seedorf G. Markham N. Abman S. H. (2012). Excess soluble vascular endothelial growth factor receptor-1 in amniotic fluid impairs lung growth in rats: Linking preeclampsia with bronchopulmonary dysplasia. Am. J. Physiol. Lung Cell Mol. Physiol. 302 (1), L36–L46. 10.1152/ajplung.00294.2011 22003089 PMC3349373

[B70] Tang J. R. Markham N. E. Lin Y. J. McMurtry I. F. Maxey A. Kinsella J. P. (2004). Inhaled nitric oxide attenuates pulmonary hypertension and improves lung growth in infant rats after neonatal treatment with a VEGF receptor inhibitor. Am. J. Physiol. Lung Cell Mol. Physiol. 287 (2), L344–L351. 10.1152/ajplung.00291.2003 15064225

[B71] Thibeault D. W. Mabry S. M. Ekekezie II Truog W. E. (2000). Lung elastic tissue maturation and perturbations during the evolution of chronic lung disease. Pediatrics 106 (6), 1452–1459. 10.1542/peds.106.6.1452 11099603

[B72] Tomita K. Saito Y. Suzuki T. Imbaby S. Hattori K. Matsuda N. (2020). Vascular endothelial growth factor contributes to lung vascular hyperpermeability in sepsis-associated acute lung injury. Naunyn Schmiedeb. Arch. Pharmacol. 393, 2365–2374. 10.1007/s00210-020-01947-6 PMC7371837 32696151

[B73] Tsikis S. T. Fligor S. C. Hirsch T. I. Pan A. Yu L. J. Kishikawa H. (2022). Lipopolysaccharide-induced murine lung injury results in long-term pulmonary changes and downregulation of angiogenic pathways. Sci. Rep. 12 (1), 10245. 10.1038/s41598-022-14618-8 35715592 PMC9205148

[B74] van der Horst I. W. Rajatapiti P. van der Voorn P. van Nederveen F. H. Tibboel D. Rottier R. (2011). Expression of hypoxia-inducible factors, regulators, and target genes in congenital diaphragmatic hernia patients. Pediatr. Dev. Pathol. 14 (5), 384–390. 10.2350/09-09-0705-OA.1 21671771

[B75] van Haaften T. Byrne R. Bonnet S. Rochefort G. Y. Akabutu J. Bouchentouf M. (2009). Airway delivery of mesenchymal stem cells prevents arrested alveolar growth in neonatal lung injury in rats. Am. J. Respir. Crit. Care Med. 180 (11), 1131–1142. 10.1164/rccm.200902-0179OC 19713449 PMC3269236

[B76] Vila Ellis L. Cain M. P. Hutchison V. Flodby P. Crandall E. D. Borok Z. (2020). Epithelial Vegfa specifies a distinct endothelial population in the mouse lung. Dev. Cell 52 (5), 617–630. e616. 10.1016/j.devcel.2020.01.009 32059772 PMC7170573

[B77] Vohwinkel C. U. Tuder R. M. (2021). Bronchopulmonary dysplasia: Endothelial cells in the driver's seat. Am. J. Respir. Cell Mol. Biol. 65 (1), 6–7. 10.1165/rcmb.2021-0145ED 33856963 PMC8320129

[B78] Voswinckel R. Motejl V. Fehrenbach A. Wegmann M. Mehling T. Fehrenbach H. (2004). Characterisation of post-pneumonectomy lung growth in adult mice. Eur. Respir. J. 24 (4), 524–532. 10.1183/09031936.04.10004904 15459128

[B79] Wallace B. Peisl A. Seedorf G. Nowlin T. Kim C. Bosco J. (2018). Anti-sFlt-1 therapy preserves lung alveolar and vascular growth in antenatal models of bronchopulmonary dysplasia. Am. J. Respir. Crit. Care Med. 197 (6), 776–787. 10.1164/rccm.201707-1371OC 29268623 PMC5855071

[B80] Wei S. C. Fattet L. Tsai J. H. Guo Y. Pai V. H. Majeski H. E. (2015). Matrix stiffness drives epithelial-mesenchymal transition and tumour metastasis through a TWIST1-G3BP2 mechanotransduction pathway. Nat. Cell Biol. 17 (5), 678–688. 10.1038/ncb3157 25893917 PMC4452027

[B81] White A. C. Lavine K. J. Ornitz D. M. (2007). FGF9 and SHH regulate mesenchymal Vegfa expression and development of the pulmonary capillary network. Development 134 (20), 3743–3752. 10.1242/dev.004879 17881491 PMC2099314

[B82] Zhao G. Weiner A. I. Neupauer K. M. de Mello Costa M. F. Palashikar G. Adams-Tzivelekidis S. (2020). Regeneration of the pulmonary vascular endothelium after viral pneumonia requires COUP-TF2. Sci. Adv. 6 (48), eabc4493. 10.1126/sciadv.abc4493 33239293 PMC7688336

[B83] Ziche M. Morbidelli L. Choudhuri R. Zhang H. T. Donnini S. Granger H. J. (1997). Nitric oxide synthase lies downstream from vascular endothelial growth factor-induced but not basic fibroblast growth factor-induced angiogenesis. J. Clin. Invest. 99 (11), 2625–2634. 10.1172/JCI119451 9169492 PMC508108

